# Nutrition Therapy in the Transition between Hospital and Home: An Investigation of Barriers

**DOI:** 10.1155/2013/463751

**Published:** 2013-12-29

**Authors:** Mette Holst, Henrik Højgaard Rasmussen

**Affiliations:** Department of Gastroenterology, Centre for Nutrition and Bowel Disease (CET), Aalborg University Hospital, Faculty of Planning, Aalborg University, 9000 Aalborg, Denmark

## Abstract

*Aims and Objectives*. This study aimed to investigate barriers for nutrition therapy in the transition between hospital and home and hereby to identify areas for potential improvements. *Background*. Though the focus on nutritional risk is improving in hospital, there seems to be less effort to maintain or even improve nutritional status after discharge and during the rehabilitation period. *Design*. Qualitative focus group interviews. *Methods*. Semistructured focus group interviews with experienced multiprofessional staff from hospital, home care, nursing homes, and general practise. The study was done in the county of Aalborg with about 280.000 inhabitants regarding homecare and general practise as well as Aalborg University Hospital, Denmark. *Results*. Interviews were generated with 41 professionals from hospital, general practise, and home care. Barriers identified between settings included the following aspects: economic, organisation, and education. The impression of professionals was that few patients are discharged with nutrition therapy, compared to who could benefit from nutrition therapy after discharge. Most often, reasons were a short in-hospital stay and lack of knowledge and interest. Moreover, lack of clinical guidelines throughout all settings, time consumption, lack of transparency regarding economy and workflows, and lack of assistance from experts regarding complicated nutritional problems were identified. *Conclusions*. Many barriers were found in hospital as well as in the community and general practise. These were most often practical as well as organizational. Improvements of clinical guidelines and instructions and improvement of knowledge and communication at all levels are needed. *Relevance to Clinical Practise*. This study emphasizes that responsibility needs to be taken for patients whom are still at nutritional risk at discharge, and even before hospitalization. Nurses and doctors in and outside hospital are in need of improved knowledge, standard care plans, and instructions.

## 1. Introduction

Nutritional risk has been described as prevalent as 20 to 80% among hospitalized patients depending on population, setting, and screening tool [1], and [2, pages 473–481]. Nutritional risk is associated with poorer outcome in many terms, including function, life quality, and mortality [3, pages 321–325], [4, pages 386–395], [5, pages 923–932], and [6, pages M741–746]. Nutritional health tends to deteriorate during hospital stay. Deterioration of nutritional status during hospital stay indicates the necessity for introducing a good nutrition plan for nutritional risk patients at discharge.

## 2. Background

Nutritional depletion during disease and hospitalization is caused by lack of appetite during acute disease, periods of fasting through clinical procedures, surgery, pain, effects of medication, and organisational aspects [7, pages 580–585], [8, pages 466–473], [9, pages 176–184], and [10, pages e22–e29]. Decreased food intake during hospitalization has significant implications, including decrease in weight, cognitive capacity, depressive symptoms, function, and increased mortality [11, pages 3299–3307], [3, pages 321–325], [1], [12, pages e30–e36], and [9, pages 176–184].

Moreover, this has to be followed up in the period of aftercare and rehabilitation, regardless whether this takes place in home, nursing home, or other care institutions [13, pages 61–67]. Studies have demonstrated that efforts on optimizing energy and protein intake at discharge and in the rehabilitation period after discharge are beneficial [11, pages 3299–3307], [3, pages 321–325], [14, pages 2239–2245], [13, pages 61–67], [15, pages 295–301], [4, pages 386–395], and [16]. A recent review found a positive effect on nutritional intake and/or nutritional status by nutritional intervention after discharge from hospital [17, pages 19–27]. These positive results were however only found in compliant patients [14, pages 2239–2245], and [3, pages 321–325]. Studies have looked into part of the trajectory of the nutrition process and found that cooperation in the care of patients with home enteral tube feeding throughout the care trajectory was influenced by the nurses' knowledge about enteral tube feeding, the discharge-planning process, and whether their responsibility was clearly distributed [18, pages 3021–3029]. In a study of care for stroke patients with eating difficulties, the investigators found that discharge summaries held poor information on care related to eating difficulties and that the language of all professionals was mostly unspecific, which led to ambiguity [19, pages 298–310].

In general practice there seems to be limited focus on undernutrition. This impression is despite the high frequency of patients at nutritional risk visiting general practice. Thus, a study of elderly patients visiting general practice in Denmark showed a frequency of 38% at nutritional risk by screening with MNA [20, pages 1028–1033]. Another study by Beck et al., found a positive impact on nutrition intake by following patients in the transition between hospital and home [16]. In this study, general practitioners were invited to visit nutritional risk patients two times with regard to follow up on nutritional and general health status in elderly medical patients after discharge. General practitioners only performed the visits in 10%, even though they were paid for this task. There has been no investigation of the reason for this absence.

When discharging a patient from hospital in Denmark with nutritional therapy such as either oral nutritional supplements or enteral nutrition, a special “nutrition prescription” is made for the patient to take to the pharmacy or to send to one of the companies who distribute nutrition for medical purposes. Investigations indicate that prescriptions for oral nutritional supplements and enteral nutrition are only to a low degree redeemed. For patients discharged with nutritional therapy, this is administered in accordance with the applicable rules for dispensing medicines. Regarding parenteral nutrition, the rules for reimbursement apply to the clinical diagnosis, which is the basis for the treatment, rather than to nutritional status.

**Figure 1:**
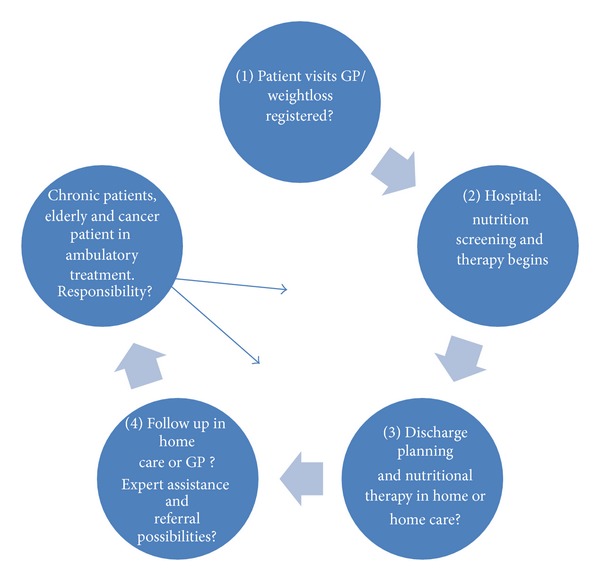
Flow chart for the patient course of the nutritional risk patient.

Many categories of subacute patients will contact the general practitioner as the first step in a long-term treatment, and unintentional weight loss seems to be the reason for many approaches to general practice. This approach may often involve referral to a course of treatment for cancer disease as well as in many chronic diseases such as chronic lung disease, and others with great impact on nutritional status. There are no available studies showing the frequency of patients whom have an initiated nutrition plan at referral to hospital for elucidation. It has however been advocated that practitioners could be urged to assess patients' nutritional status throughout the disease course and intervene if necessary [21, pages 16–21].  Figure 1 shows the nutritional course for the nutritional risk patient.

The underlying understanding of this study is that the nutritional course in patients starts from the time a patient contacts the general practitioner and ends when the patient has regained health and is no longer at nutritional risk by screening and assessment.

The present study aimed to investigate and describe eventual barriers for nutrition therapy for nutritional risk patients in the transition between hospital and home in a Danish University Hospital.

## 3. Methods

### 3.1. Design

Semistructured focus group interviews were generated in monodisciplinary sessions in hospital, with community nurses and social care assistants in nursing homes and home care and by general practitioners, respectively [22, 23].

### 3.2. Data Collection

The groups were invited to be active participators in an open recorded indebt conversation together with mono-professionals from the same setting (hospital, community or general practice) about the eventual barriers for nutrition therapy in the transition between hospital and home. The first author, who was experienced with the method and nutritional aspects, conducted the interviews. Interviews were recorded on Olympus Digital Recorder DS-75.

The audio-recorded data were listened to thoroughly twice by the interviewer and assistant, respectively. In the second listening, sentences and passages of meaning were transcribed verbatim. The transcribed data was reread in common between the investigators for understanding.

All material was analysed in the phenomenological hermeneutic framework. Afterwards the analysed data were discussed and concluded upon together [24, page 29].

### 3.3. Interview Guide

The semistructured interview guide was limited to keywords, to ensure that the pathway of the nutritional risk practice between sectors was held. Keywords were *Nutritional risk, relevance, admission, discharge, nutrition therapy, external communication, and documentation*. The interviews were opened by the question “Is it at all relevant to talk about nutritional risk of patients in the transition between sectors?”

### 3.4. Sample

The sampling procedure for recruiting participants was informed by a purposive sampling strategy [25]. In the hospital, which was a university hospital with 780 beds and all specialties, the interviews were announced through the multiprofessional nutrition teams. The participants volunteered. Nursing staff from the community was selected through the canals of a member of an interest group for nutrition, who was a leader in the primary nursing organization of the community. The general practitioners (GPs) and consultation nurse were selected by the head of the GP steering committee in the region.

### 3.5. Data Analysis

Data analysis was an iterative and back-and-forth process. This involved reading and rereading the transcribed data with the aim of being immersed in the data and getting a sense of the group discussions and meanings [26, pages 655–660], and [27]. This was followed by noting reflections and sorting the data by identifying similar phrases and distinct differences within and between groups. Each focus group interview was explored, followed by cross-case analysis to identify similarities and differences within the themes across all of the data. The data were then compared and contrasted. The final step involved interpretation and understanding of data, and the identified categories were connected in patterns of major themes including their related subthemes.

We considered the interactions between participants through our analysis by examining the negotiations, agreements, disagreements, and accounts that were used in the discussions [27], [26, pages 655–660], and [23]. The results described were general for the interview, and thereby consensus statements of the group sessions. Single opinions, that could not be shared or added to by the other participants in the interviews, were few and have been excluded in this paper.

### 3.6. Ethics

The study aimed at investigating barriers equally in all relevant professionals on a voluntary basis. Patients and relatives have not been heard in this study.

## 4. Results

The dataset included 41 participants in eight focus group interview sessions. One interview was a single interview. The others included four to eight participants. Three interviews were generated in hospital.

Five interviews were generated with primary sector staff including GPs. Two of these interviews took place in a meeting room in the hospital. The others took place in meeting rooms at the participant's own settings, general practice, and community care offices. The interviews lasted from 25 minutes (the single interview) up till two hours.

In the following, the results from each interview will be described individually under the headline of profession and place of work.

### 4.1. Hospital Nurses

Six nurses from different specialties: hematology, infectious disease, orthopedic surgery, pulmonary disease, and gastroenterology surgery participated in the interview. The organizational levels represented were basic nurse to head nurse, and the level of clinical experience ranged from a few to 35 years.

The main message from the hospital nurses was that many patients who would benefit from a nutrition plan at discharge are probably not discharged with such a plan. Many reasons formed the basis of this opinion, which was not proudly stated. The results below have been structured respectively in “internal barriers,” which were barriers found within the hospital setting, and “external barriers,” describing the barriers found in the collaboration with the community. Generally, the nurses had never experienced a patient who was admitted to hospital with a nutrition plan initiated in general practice.

### 4.2. Internal Barriers

A short length of stay was also given as a reason for simply not always having the time to make a thorough nutrition plan, even though the need for one was justified by nutrition screening, which was done routinely in all patients on admission. The nurses found that due to factors of lack of knowledge and experience among their colleagues, nutrition therapy might not be initiated at all in some patients. For the same reasons, they were of the impression that an eventual nutrition plan would not always be evaluated and continued at discharge. The group had a common understanding that the attitude towards nutrition practice was individual, and that this was to a high degree linked to knowledge and experience. They found that some nurses were more designated to follow the hospital guidelines for nutrition than others. This was also found to be the case for the doctors, who had the responsibility for prescribing nutritional treatment if this included enteral or parenteral nutrition.

Nutrition planning after discharge was found to be resource-demanding, especially regarding parenteral nutrition. These caring procedures involved teaching the patient and spouse regarding care for central lines and handling of the parenteral nutrition, as well as many other procedures such as ordering of other necessary articles. These procedures were seldom done, and not even the most experienced nurses remembered the many workflows from time to time. Some had used the possibility of calling the nurses at the short bowel unit; however, these were most often just able to give advice by phone. Furthermore, there was a lack of transparency in relation to economy, especially concerning the responsibility for reimbursement of parenteral nutrition after discharge. All agreed that palliative care patients were reimbursed and that patients with short bowel syndrome had a special DRG-code for reimbursement. However, there was uncertainty about all other diagnosis. This led to many considerations regarding the cost for an individual department, and was the reason why the doctors would often discontinue the therapy at discharge, or be reluctant to start it because of a short-term stay.

### 4.3. External Barriers

When nutrition plans were actually in focus for patients at discharge, many barriers were found before a sufficient plan could be performed. In the discharge procedure involving nutrition, the hospital nurses found that lack of uniformity and transparency for community procedures and possibilities were obstacles. The home care nursing was organized in smaller units, and the patients were assigned to a unit after their home address. The nurses, however, found that the services in these units differed and that one unit could provide much more care than the other, for instance regarding assistance in meal situations. More nurses had experienced reluctance from some home care units to care for patients with enteral nutrition through a nasogastric tube. Regarding parenteral nutrition, a very large discrepancy was found concerning knowledge, skills, and the general management. If the patient or spouse were not able to handle the parenteral nutrition care themselves, this would have to be done by home care nurses. Teaching home care how to handle parenteral nutrition and line care was considered the task of the hospital, if the patient was discharged with home parenteral nutrition. This education of home care nurses was done by the nurses taking care of the individual patient in the hospital department, and who was not necessarily familiar with education strategies for groups, and who was often also new in the field themselves. Home care could in practice then ask for as many education sessions as they liked until they found themselves capable of providing the patient with safe care. The experience of the interviewed group was that this could take from one session up to four sessions within a three week period. This might extend the discharge procedure.

### 4.4. Hospital Doctors

Four doctors, including the following specialties: medical gastroenterology, hepatology, oncology, and surgical gastroenterology, participated in the interview. Two of the doctors were experienced staff specialists and two were consultants.

The doctors likewise found that there were probably fewer patients who were prescribed with nutrition therapy at discharge, as could benefit from it. They, however, did not find themselves very much involved in nutrition at discharge, but were likely to believe that their involvement was partly a matter of interest, and that doctors' involvement in nutrition in the overall patient course from admission to discharge, would to some point rely on personal interest. Their overall task was to prescribe nutrition therapy, to write the prescription, and to give notice back to the patients' general practitioner. They were aware that the nurses had a lot of work to do around the discharge of patients with artificial nutrition and found that they might sometimes lay a pressure on the nurses to send the patients home before the nurses actually have had the time to finish the nutrition care plan. Economy was another problem regarding parenteral nutrition. The doctors found themselves in a pinch between the departments' financial terms and the terms of the patient. If the patient was able to eat, they would rarely prescribe the patient with parenteral nutrition if the patient was ready for discharge within a few days. The doctors would then encourage the patient to eat and take supplements instead.

### 4.5. Hospital Dieticians

Four dieticians took part in the interview. These dieticians represented a broad variation of specialties, including gastroenterology, oncology, kidney disease, and neurology. Their overall primary focus was nutritional therapy for nutritional risk patients.

The dieticians were involved with patients before discharge when referred from doctors. Their primary task was to assist with the planning of nutritional therapy in hospital. They would mostly assist with the planning of which type of nutrition support to give to the patients, and how much. If the patient was to be discharged with a special diet recommendation including nutrition supplements, the dieticians took care of giving advice to the patients, and sometimes spouse. None of them found themselves very often involved in artificial nutrition therapy at discharge, and if they were, the nurses would take care of all the practical handling and contact to the home care. The dieticians sometimes saw the patient in the outpatient clinic after discharge, if a planned nutrition therapy was established, that is, in oncology, Crohn's, or pancreatic disease patients, as well as in patients on dialysis. If the patient was discharged without any follow-up for their disease, the dieticians rarely had the opportunity to follow the patients' nutritional status.

### 4.6. Community Home Care Nurses

For practical reasons, the community care nurses were interviewed in two sessions. The community nurses interviewed came from two of the largest population areas of the region. In one session, there were four participants, and in the other, five participants. The two interviews were analyzed separately; however, the findings were comparable, and the results are thus described together.

The home care nurses found themselves quite well prepared for taking over practical issues of patients' sent home with nutrition therapy, if they were given sufficient information from the hospital. However, they often found this was not the case. They found that they were not given any information about the reason for discharge with a nutrition plan, neither the goal, that is, when or how to follow up, and when to change the nutrition therapy. They often found that important information was not given from the hospital, and when they contacted the hospital, they only rarely got useful information about the patient's nutritional recommendations. They agreed that some of the problems could relate to a communication problem between the administrative level and the basic home care nurses directly involved with the patient care. They received no useful written information about the patient's nutrition in the IT-generated system, and only incidentally the patient brought home a printed letter of information.

The home care nurses had no possibilities to consult anybody but the patients' general practitioner, if the patient did not gain weight, gained too much weight, or was not able to comply with the prescribed nutrition therapy.

Practical issues as well as economical issues were found to be very important. If a patient was sent home with a nasogastric tube, there was a safety rule that the nurses themselves had to provide the patient with bolus feeding, and that continuous feeding, that is, overnight, was not allowed. This task was not to be given to less educated staff. Time spent on this, including many times of daily transportation, was the main reason why the nurses were reluctant to take patients home with a nasogastric tube. Furthermore, it was advocated that a nasogastric tube was bothersome to the patient, and made the patient look very sick. They preferred percutaneous tubes; however, the nursing staff had no knowledge or reflection of the risks associated with the insertion and daily use of these tubes.

Regarding guidelines and knowledge of handling the different nutritional strategies, including different tube-types and line care, there were no updated guidelines. The existing documents for practice were printed, and generally there were no common documents or recommendations for nutritional care as such.

If the nurses had trouble with handling nutrition therapy in a patient, and the GP was not expected to be of help, the nurses would contact a nutrition company for assistance with teaching or practical issues.

The home care nurses found that they were well educated regarding energy and protein dense meals and nutritional supplements, but in general they found that it was difficult to motivate patients to eat and that they were much dependent on their less educated colleagues to take care of this part, since the nurses only came to the house more seldom and for other special tasks.

### 4.7. Social Care Assistants of Home Care and Nursing Homes

In this interview, there were eight participants. These were employed in the same organization; however, some were mostly assigned to work in nursing home, and some moreover in the patient home care setting.

Social care assistants were mostly involved with practical issues around helping with meals and enteral nutrition through PEG-tubes. They found that they were well educated for helping patients with eating as well as serving and giving nutritional advice for patients with an insufficient food intake. They served nutritional supplements for patients who had these prescribed, and would encourage their clients to take these. They found that they did not have sufficient information about the goal for weight gain or other monitoring from the hospital when they received clients back from hospital, and they did not do monitoring on a routine basis unless this was requested. They found that the assignment of responsibility regarding nutritional care between them and the homecare nurses was unclear, especially if the nurse was involved only in the nutritional therapy, to a low degree or not involved in the client.

Sometimes they would observe that a client had lost weight, and they would weigh the client if possible. In this case, they would contact the home care nurse if such was available, and/or talk to the relatives about consulting the general practitioner.

They were given education in enteral nutrition and nutritional supplements by the consultant from the nutrition company. They rarely took contact to the community dietician, as they found that some controversy existed between the community dietician and the company consultant about what was to be done. They found that this was difficult to handle.

### 4.8. General Practitioners

Nine general practitioners (GPs) from eight different private consultations took part in the interview, which was held in one of the consultations.

The general practitioners found, that they in general involved themselves too little in undernutrition, and that their knowledge and practice for clinical nutrition were vague. Their only obvious treatment option was giving nutritional supplements or referral to home care nurses or to their consultation nurse, who mostly took care of routine annual investigations for chronic disease patients and elderly. Their primary focus was investigation for disease, and if the patient had lost weight, they would regard it as taken care of by the hospital, to which they referred the patient for further investigation. They would always inform the hospital of the weight loss at referral. Otherwise, they moreover regarded nutrition in the perspective of overweight and prevention.

Only one of the nine GPs was aware that they could in fact refer elderly and chronic patients to a community dietician for nutrition therapy.

### 4.9. Consultation Nurse in General Practice

The consultation nurse interview was decided since it emerged in the general practitioner interview, that especially patients who suffered from chronic diseases, including chronic pulmonary disease and diabetes, were routinely seen on an annual basis in nurse consultations under the auspices of general practitioners. She was also able to do home calls if the patient was not able to come to the consultation. She was never involved with patients during investigation for disease.

The consultation nurse found herself quite well educated towards talking to the patients about energy, and protein supplementation, and energy dense meals. In all consultations, she would weigh patients and talk to them about nutrition intake. She found however that she missed someone to interact with, when a patient at nutritional risk did not gain weight as planned or if the patient had continued functional decline despite the nutritional therapy.

### 4.10. Themes

**Figure 2:**
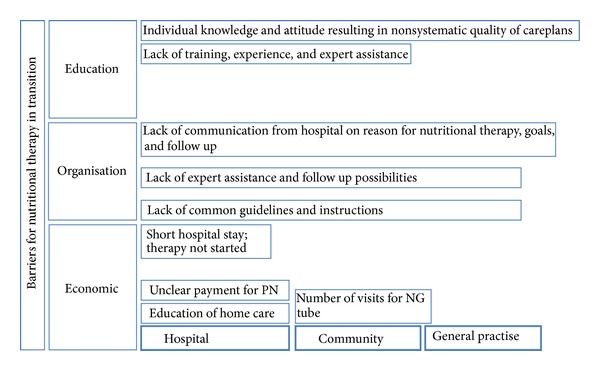
Barriers for nutritional therapy in the transition between hospital and home.

The findings in the interviews generated three themes of barriers: education, organization, and economy. These themes (Figure 2) were found in all professions, settings, hospital, community, or general practise.

## 5. Discussion

This study aimed to identify barriers for good nutrition practise in the transition of patients between hospital and home, and hereby to identify improvement potentials. These interviews with 41 involved parties revealed improvement potentials in all groups as well as in-between sectors. Overall, the findings in the interviews generated three themes of barriers: *education, organization, and economy.* In this section, the results are described and discussed according to these themes.

### 5.1. Economic

All groups agreed that there were probably more patients who could benefit from nutrition therapy after discharge. Doctors, nurses, and dieticians in hospital shared the same view, that some patients did simply not have nutrition plans made, because of the short time to initiate this before discharge. However, all patients with a suspected in-hospital stay of 48 hours should be screened on admission, according to regulations from the Danish National Board of Health (RW.ERROR-Unable to find reference: 326). Thus, the identification of patients at nutritional risk should be done in all patients. Time to make a relevant nutrition plan should be allocated, even though the monitoring and follow up part might be handed over to other parties because of early discharge. It does, however, seem somewhat ambiguous that screening for nutritional risk is highly prioritized and has been included in the accreditation criteria's, but the time and effort to make a thorough nutrition plan to follow until patients have gained health is not prioritized. Lack of time as well as economical aspects were also in focus, when establishing parenteral nutrition in patients after care. It was striking that doctors and nurses would spent so much time and economic resources on the treatment of patients while at hospital, but did not take responsibility for the patient to gain nutritional status and general health in the period of convalescence, thus beneficial [11, pages 3299–3307], [3, pages 321–325], [14, pages 2239–2245], [13, pages 61–67], [15, pages 295–301], [4, pages 386–395], and [16]. However, it does not seem rational that every doctor and nurse should have these conflicts in their daily effort to treat the patients. These problems should be addressed as clinical instructions and politics from the central hospital management, if not given clearly from the National Board of Health.

### 5.2. Education

This study shows that knowledge, standards, and clear updated instructions are clearly needed by all professionals, since individual handling and interest were seen as defining whether a patient was given a nutrition plan at discharge or not. The same applied for home care nursing, where evidence-based updated standards and instructions could, if applied, ensure the safe care for patients. Nurses only rarely discharged patients with parenteral nutrition, and especially they felt a lack of competence regarding education of the nurses in the community, whom also for some areas, showed reluctance to make this task go smoothly. Knowledge about nutritional aspects to the discharge planning process and the distribution of responsibility between professionals were regarded as important, as seen in an earlier study [18, pages 3021–3029]. Neither the hospital nurses, nor the community had access to updated evidence-based care plans and instructions regarding discharging patients with parenteral nutrition at home.

Also the GPs claimed to lack knowledge and expertise of clinical nutrition. Former studies have also found that these issues are given very little notice [21, pages 16–21], [16], and [11, pages 3299–3307]. GPs found that the consultation and home care nurses had more knowledge concerning giving advice to patients about nutrition. Meanwhile, the general practitioner is often the first contact for patients regarding a diagnose-finding process, as well as the only contact when patients still at nutritional risk are discharged without contact to home care nurse or a specific convalescence. A recent study from the Netherlands showed that the contact information from hospital physician to GP was very poor [28]. Another Danish study showed, that even when GPs were involved in a discharge project regarding nutritional status and care, they only, to a very low degree, fulfilled the monitoring visits [16].

The present study lacks details of the content of education, knowledge, and tools needed in general practise. Furthermore, evidence to show the efficacy of education initiatives is sparse.

### 5.3. Organisation

The quite clear recommendations from the Danish National Board of Health should ensure the trajectory for patients at nutritional risk, including the distribution of information to other caregivers, when the patient is transferred between sectors (RW.ERROR-Unable to find reference: 326). The Danish National Board of Health 2008, a guide for doctors, nurses, health care assistants, auxiliary nurses and clnical dietitians- Screening and treatment of patients at nutritional risk http://www.sst.dk/publ/Publ2008/CFF/ernaering/Screening_of_patients_at_nutritional_risk_Danish_NBH_2008.pdf. According to the interviews in the present study, this information was not or was insufficiently given in the majority of cases. From the hospital view, there was a lack of transparency of where and how to place information, in order to reach the relevant person, and furthermore, local variety for what and how much care and service could be provided. This lack of transparency, allocation of responsibility, and structure for the organisation of clinical nutrition seem to be of importance [10, pages e22–e29]. Unified efforts have been seen to improve patient outcome and satisfaction in patients, relatives, and healthcare staff [28].

None of the hospital staff, including oncology and department for lung disease, had ever received a patient with a nutrition care plan from the general practitioner. This is despite the fact that oncology patients may often see their GP because of involuntary weight loss, and lung disease patients are seen at least once a year by the consultation nurse. The GPs were moreover not aware of the possibility to actually refer patients to a community dietician, and the consultation nurse lacked someone to consult about nutrition related problems.

Even though the community dietician would have been commonly known and used, discussions of complicated patients concerning nutritional problems were needed with specialists in clinical nutrition both from the dietician, the general practitioner, consultation, and home care nurses as well as between parties. These patients include cancer patients, patients in convalescence, elderly with multifactorial diseases, and patients with chronic diseases.

Education and a multiprofessional expert centre working trans-sectoral between hospital, community, and general practise, in order to supervise clinical staff, take care of evidence-based guidelines in a unified system, and see complicated patients, might be a solution, as seen in former studies, however, in selected patient groups [29].

In summary, this study showed that barriers are seen for practising clinical nutrition in the transition between hospital and home, as well as in home and general practise. These barriers regarded the education of staff, including staff resources for ensuring proper education in the transition between hospital and home, and clarifying of financial problems for parenteral nutrition after discharge. An improved organization with regular flow of information, as well as clear chains of command was requested.

Relevance to clinical practice, consider the following.

- The overall impression was that not nearly as many patients are discharged with a nutrition plan, as there are patients who could benefit from one.
- Barriers are found in hospital, community, and general practice. Improved knowledge, standard care plans, and instructions throughout the organisations are needed.
- Financial responsibility from doctors may influence clinical decision-making negatively, if economic consequences, knowledge, guidelines, and instructions are not in place.
- A multiprofessional expert centre for clinical nutrition working between the sectors is suggested.
